# Accelerating Multiple Compound Comparison Using LINGO-Based Load-Balancing Strategies on Multi-GPUs

**DOI:** 10.1155/2015/950905

**Published:** 2015-10-13

**Authors:** Chun-Yuan Lin, Chung-Hung Wang, Che-Lun Hung, Yu-Shiang Lin

**Affiliations:** ^1^Department of Computer Science and Information Engineering, Chang Gung University, Taoyuan 33302, Taiwan; ^2^Department of Computer Science and Communication Engineering, Providence University, Taichung 43301, Taiwan; ^3^Department of Computer Science, National Tsing Hua University, Hsinchu 30013, Taiwan

## Abstract

Compound comparison is an important task for the computational chemistry. By the comparison results, potential inhibitors can be found and then used for the pharmacy experiments. The time complexity of a pairwise compound comparison is *O*(*n* ^2^), where *n* is the maximal length of compounds. In general, the length of compounds is tens to hundreds, and the computation time is small. However, more and more compounds have been synthesized and extracted now, even more than tens of millions. Therefore, it still will be time-consuming when comparing with a large amount of compounds (seen as a multiple compound comparison problem, abbreviated to MCC). The intrinsic time complexity of MCC problem is *O*(*k* ^2^ *n* ^2^) with *k* compounds of maximal length *n*. In this paper, we propose a GPU-based algorithm for MCC problem, called CUDA-MCC, on single- and multi-GPUs. Four LINGO-based load-balancing strategies are considered in CUDA-MCC in order to accelerate the computation speed among thread blocks on GPUs. CUDA-MCC was implemented by C+OpenMP+CUDA. CUDA-MCC achieved 45 times and 391 times faster than its CPU version on a single NVIDIA Tesla K20m GPU card and a dual-NVIDIA Tesla K20m GPU card, respectively, under the experimental results.

## 1. Introduction

A new drug to market usually costs a lot of time for the research and the development and invests a huge amount of money. After decoding the human genome, the molecular biology and the proteomic fields make a remarkable advance; people understand more clearly the disease generation and the disease mechanism. Computer-Aided Drug Design (abbreviated to CADD) [1] becomes an emerging research field, and it is helpful to improve the efficiencies of drug design and development. CADD is a kind of approaches, named rational drug design. Rational drug design is based on the structure, the property, and the mechanism. There are two major approaches: structure-based approach and ligand-based approach. Researchers use these two approaches to develop drugs depending on different drug design strategies. Structure-based approach mainly uses the techniques of docking [2, 3] and de novo ligand design [4], and the ligand-based approach uses the techniques of QSAR [5] and Pharmacophore [3, 6] mostly. Based on these two approaches, potential inhibitors can be found for the target genes or proteins. After that, in general, these potential inhibitors will be used to compare with the compound databases, such as ZINC [7], PubChem [8], and GDB-13 [9], in order to find other compounds with similar structure. These similar compounds may be helpful for shortening the subsequent synthesis procedures of potential inhibitors. The potential inhibitors and similar compounds are then used for the pharmacy experiments to test the biological activity, toxicity, and so forth. For well-known drugs, the compound comparison also can be used to find the generic drugs.

Therefore, compound comparison has been an important and commonly used task for the computational chemistry. Many algorithms [10] have been proposed to do the compound comparisons in the past. The Tanimoto coefficient is one of the most popular measurements between two molecules (compounds) due to its computational efficiency and its relevance to biological profile [11, 12]. In most of the works, molecules can be represented as fingerprints and SMILES, and then the work of compound comparison can be seen as the string comparison problem. For example, the LINGO method [13] proposed by Vidal et al. is a simple algorithm to compute the chemical similarity between two SMILES-based molecules, and it has demonstrated the accuracy comparable to fingerprint methods [14]. For a pairwise compound comparison, the time complexity is *O*(*n* ^2^), where *n* is the maximal length of compounds. In general, the length of compound is short (e.g., tens to hundreds) and the computation time is small. However, it will be time-consuming when compared with a large amount of compounds (denoted by a multiple compound comparison problem, abbreviated to MCC), such as ZINC database with more than 60 million compounds and GDB-13 database with more than 970 million small molecules. The intrinsic time complexity of MCC problem is *O*(*k* ^2^ *n* ^2^) with *k* compounds of maximal length *n*. Hence, how to accelerate the MCC problem is an important issue.

It is a feasible direction to apply parallel technologies and multicore devices into the above issue. The feasibility of using massive computational devices to enhance the performance of many programs has received considerable attention in recent years, especially for many-core devices, such as FPGAs [15–17] and Cell/Bes [18–20]. Current high-end graphics processing units (abbreviated to GPUs) [21, 22], which contain up to thousands of cores per chip, are widely used in the high performance computing community. As a massively multithreaded processor, GPU expects the thousands of concurrent threads to fully utilize its computing power. The ease of accessing GPUs by using General-Purpose computing on Graphics Processing Units (abbreviated to GPGPU) such as Open Computing Language (abbreviated to OpenCL, https://www.khronos.org/opencl/) and compute unified device architecture (abbreviated to CUDA [23]), as opposed to graphic APIs (as OpenGL), has made the supercomputing available widely. CUDA uses a new computing architecture referred to as single instruction multiple threads (SIMT), which differs from Flynn's classification [24]. Importantly, the computing power and the memory bandwidth for modern GPUs have made porting applications more possible.

For the MCC problem, it can be done to compare a compound with a set of compounds (denoted by one to all, abbreviated to O2A) or to compare two sets of compounds (denoted by all to all, abbreviated to A2A). Several GPU-based parallel algorithms were proposed in the past. For example, Haque et al. proposed a GPU-based parallel algorithm, called SIML (full name is Single-Instruction Multiple-LINGO [25]), to calculate the Tanimoto coefficients between SMILES-based molecules. The SIML algorithm is designed based on the LINGO method, and its GPU implementation is over 30 times faster than its CPU version. Ma et al. presented a parallel algorithm [26] to calculate the Tanimoto coefficients for MCC problem between molecular fingerprints on GPUs. The experimental results showed that the implemented program achieved 39 times faster than Sybyl Database Comparison program that runs on CPUs and 10 times faster than other GPU-based programs [25, 27]. However, it is unfair to compare their algorithm based on the fingerprints representation with other algorithms based on the SMILES representation. The reason is that the computation is different for these two representations. Among these works, it is still insufficient for designing a GPU-based algorithm for MCC problem. At first, most of these works were focused on single-GPU card. Second, their tests were based on old GPU cards and CUDA capability. Third, they did not apply various load-balancing strategies into their works and then discuss their effects. A suitable load-balancing strategy can accelerate the computation speed on single- and multi-GPU cards.

Hence, in this paper, we propose a GPU-based algorithm for MCC problem (O2A and A2A) on single- and multi-GPUs, called CUDA-MCC. As the work [25], CUDA-MCC is also based on the LINGO method to calculate the Tanimoto coefficients between SMILES-based molecules. Four LINGO-based load-balancing strategies were also applied into CUDA-MCC by considering the LINGO score, LINGO number, LINGO length, and LINGO magnitude, respectively. CUDA-MCC was implemented by C+OpenMP+CUDA for single- and multi-GPU cards. The experimental tests were done on single NVIDIA Tesla K20m GPU card and dual-NVIDIA Tesla K20m GPU cards, and the experimental results showed that CUDA-MCC can achieve 45 times and 391 times faster than its CPU version on the above experimental environments, respectively.

## 2. Background Knowledge

The LINGO method is to model a molecule as a collection of substrings of SMILES representation (seen as a string). Thus, a SMILES string is fragmented into all substrings with length of *q* by using the sliding window scheme. These substrings are stored as a set of *q*-Lingos. In addition, the information of each *q*-Lingo is also stored in order to do the following comparison. For example, as shown in the literature [25], the score, length, number, and magnitude of each *q*-Lingo are stored. When doing the pairwise compound comparison, two sets of *q*-Lingos from two molecules are used to find the same Lingos. The number of the same Lingos is then used to calculate the Tanimoto coefficients. The details of LINGO method can be found in the literature [13]. Therefore, there are four LINGO-based load-balancing strategies by considering the score, length, number, and magnitude of each molecule represented by *q*-Lingos.

CUDA is an extension of commonly used programming languages, such as C/C++, in which users can write scalable multithreading programs for various applications. In general, the CUDA program is implemented in two parts: host and device. The host part is executed on CPU, and the device part is executed on GPU. The function executed on the device part is called a kernel. The kernel can be invoked as a set of concurrently executing threads. These threads are grouped into a hierarchical organization which can be combined into thread blocks and grids. A grid is a set of independent thread blocks, and a thread block contains many threads. The size of grid is the number of thread blocks per grid, and the size of thread block is the number of threads per thread block.

Threads in a thread block can communicate and synchronize with each other. Threads within a thread block can communicate through a per thread block shared memory, whereas threads in the different thread blocks fail to communicate or synchronize directly. Besides shared memory, five memory types are per grid private local memory, global memory for data shared by all thread blocks, texture memory, constant memory, and registers. Of these memory types, constant memory and texture memory can be regarded as fast read only caches; the fastest memories are the registers and shared memory. The global memory, local memory, texture memory, and constant memory are located on the GPU's memory. Besides shared memory accessed by a single thread block and register only accessed by a single thread, the other memory types can be used by all of the threads. The caches of texture memory and constant memory are limited to 8 KB per streaming multiprocessor. The optimum access strategy for constant memory is all threads reading the same memory address. The cache of texture memory is designed for threads to read through the proximity of the address in order to achieve an improved reading efficiency. Fermi and Kepler architectures have real configurable L1 per streaming multiprocessor and unified L2 caches among streaming multiprocessors. Hence, L2 caches can be accessed by global memory and each streaming multiprocessor can use the L1 caches and shared memory.

The basic processing unit in NVIDIA's GPU architecture is called the streaming processor. In Fermi and Kepler architectures, the basic processing unit is called CUDA cores. Many streaming processors perform the computations on GPU. Several streaming processors can be integrated into a streaming multiprocessor according to various architectures, such as 32 and 192 streaming processors per streaming multiprocessor for Fermi and Kepler architectures, respectively. While the program runs the kernel, the device schedules thread blocks for the execution on the streaming multiprocessor. The SIMT scheme refers to threads running on the streaming multiprocessor in a small group of 32, called a warp. The warp scheduler simultaneously schedules and dispatches instructions.

## 3. Method

**Figure 1:**
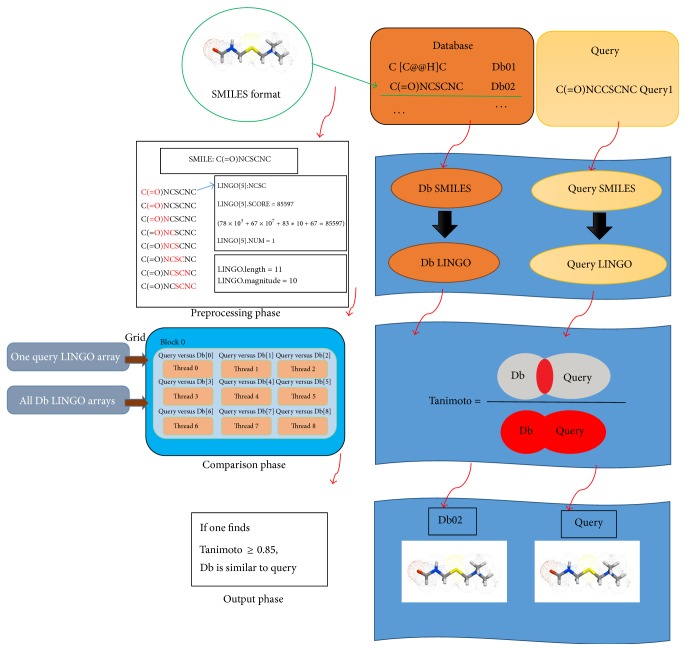
The flowchart of three phases in CUDA-MCC.

In CUDA-MCC, the goal is to compare two sets of compounds (A2A) listed as* Query* and* Database* at first and then find the compounds in* Database* with more than 0.85 Tanimoto coefficients for each compound in* Query*. CUDA-MCC also can be used to do the O2A comparison when the* Query* is with only one compound. For each compound in* Query* and* Database*, it should be fragmented into a set of *q*-Lingos mentioned in Section 2, respectively. Grant et al. [14] have demonstrated that setting *q* = 4 can have the best performance in various cheminformatics applications. Hence, in CUDA-MCC, the *q* is set to 4. Since this procedure is only done once, a* preprocessing phase* is designed in CUDA-MCC to do this procedure on CPU. After this phase, the information of* Query* and* Database* is transferred from CPU to GPU, and then a GPU implementation of* comparison phase* is designed in CUDA-MCC in order to accelerate the computation speed. Finally, the Tanimoto coefficient of each pair of compounds is stored in a result array on GPU, and then this result array will be transferred from GPU to CPU. All the compounds in* Database* with more than 0.85 Tanimoto coefficients for each compound in* Query* are reported in the* output phase* on CPU. The flowchart of three phases in CUDA-MCC is shown in Figure 1. In Figure 1, the first three processes for* Query *and* Database* are the* preprocessing phase*, followed by the* comparison phase*, and* output phase* which is the last phase. The details of these three phases are described below.

### 3.1. Preprocessing Phase

**Figure 2:**
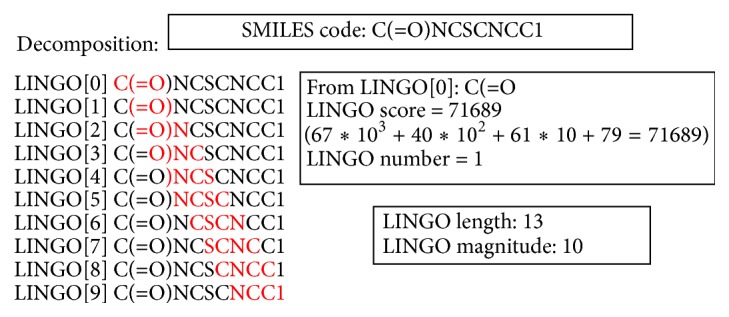
An example of a SMILES code in the* preprocessing phase*.

This phase can be divided into three parts:* reading files*,* Lingo construction,* and* Lingo sorting*. In the* reading files* part, there are two databases* Query* and* Database,* as input files should be read from the disk to memory space on CPU. For these two databases, the compounds are stored in two-dimensional string arrays, *Q* and *Db*, respectively. In *Q* and *Db*, each compound is represented as the SMILES code, as shown in Figure 2.

After that, in the* Lingo construction* part, each SMILES code (as a string) is fragmented into a set of 4-Lingos (as substrings) by using the sliding window scheme with an offset 1 on CPU. For a 4-Lingo, it will be transformed into a 32-bit integer (called** LINGO score**) according to the ASCII code table in order to accelerate the comparison in the* comparison phase*. For a SMILES code with length *l*, it can be fragmented into *l*-3 4-Lingos and this value *l* is called** LINGO length**. Hence, for a SMILES code, a temporary one-dimensional integer array and an integer variable are used to store the LINGO score of each 4-Lingo and LINGO length, respectively. Since there are possible repeats (for each 4-Lingo) in this array, the number of repeats for each 4-Lingo is calculated. An integer variable,** LINGO number**, for each LINGO score, is used to record the times of a 4-Lingo appearing in this compound; for example, for a 4-Lingo, 1 represents only once and two or more represents the repeats. The number of 4-Lingos without repeats is also calculated and then recorded in an integer variable,** LINGO magnitude**. For a compound, there are four LINGO types: LINGO scores, LINGO length, LINGO numbers, and LINGO magnitude. An example of a SMILES code in the* preprocessing phase* is shown in Figure 2. In order to simplify the figure, the remaining LINGO scores of nine 4-Lingos are omitted in Figure 2. In this case, the LINGO number is 1 for each 4-Lingo. The LINGO length is 13 and the LINGO magnitude is 10 which is equal to the number of 4-Lingos.

For a pair of compounds, the Tanimoto coefficient is calculated according to the number of similar 4-Lingos. Therefore, in order to accelerate the computation and reduce the unnecessary comparisons in the* comparison phase*, for each compound, its LINGO scores are sorted by using the quick sort algorithm on CPU in the* Lingo sorting* part. Therefore, for* Query* and* Database*, a two-dimensional integer array is used to store the sorted LINGO scores of each compound (4-Lingos); a two-dimensional integer array is used to store the LINGO numbers of each 4-Lingo; a one-dimensional integer array is used to store the LINGO lengths of each compound; a one-dimensional integer array is used to store the LINGO magnitudes of each compound. These arrays mentioned above can be packaged in a complex structure array (LINGO constructor). The information was stored in a complex structure array for* Query* and* Database*, respectively, as shown in Figure 1.

The pseudocodes of LINGO constructor, LINGO number, LINGO score, LINGO magnitude, and LINGO length are listed  in Pseudocode 1.

In this paper, we focused on the* comparison phase* implemented on GPU. Therefore, the above three parts are all done on CPU in order to simplify the problem in CUDA-MCC. In practice, the second and third parts also can be implemented on GPUs. When the second part is implemented on GPU, the memory usage should be considered. Since the number of LINGO magnitudes is unknown after the* reading files* part, the fixed (large) memory space of structure array in each compound should be allocated on GPU at first. By this way, the memory usage may be large and most memory space is wasted. Four LINGO types of each compound can be calculated by a thread; hence, thousands of concurrent threads can be used to process these compounds quickly. After that, the wasted memory space could be removed by the memory reallocated procedure and the prefix sum algorithm. It is easy to reallocate the previous structure array into a new one on GPU by all threads according to the indices, which is computed by GPU-based prefix sum algorithm published in the past. For the implementation of the third part on GPU, many GPU-based sorting algorithms have been proposed in the past. These GPU-based sorting algorithms can be modified to sort these LINGO scores quickly on GPU. In the following experimental tests, the computation time of* preprocessing phase* is not included in the time analysis; it only includes the time of* comparison phase* and* output phase*.

### 3.2. Comparison Phase

Before designing the* comparison phase* in CUDA-MCC, how to assign the comparison tasks on GPUs should be discussed. To compare two sets of compounds (A2A) can be seen as to compare a compound with a set of compounds repeatedly (O2A). Therefore, in CUDA-MCC, a compound in* Query* will be used to compare with all compounds in* Database* by all threads on GPUs when executing the kernel function once. In a thread block, each thread is used to compare a compound in* Query* with one of the compounds in* Database*. Hence, the computing workload of each thread in a thread block should be equal in order to achieve the high performance. For multi-GPUs, the computing workload of each GPU card should also be the same.

A load-balancing strategy can be used to accelerate the computation speed for single- and multi-GPU cards. However, how to estimate the computing workload of a comparison is a problem, since only a compound in* Query *is used when executing the kernel function once. The computing workloads only need to consider the compounds in* Database*. The LINGO length and LINGO magnitude can be used directly as the measurements of computing workloads. The sums of LINGO scores and LINGO numbers can be calculated as shown in the pseudocodes of* preprocessing phase*, respectively, and then they are used as other measurements of computing workloads. Four LINGO-based load-balancing strategies are applied into CUDA-MCC by considering these four LINGO types. As mentioned in the* preprocessing phase*, the list of compounds in* Database* (structure array of* Database*) is sorted on CPU. In the* comparison phase*, on single-GPU, the structure arrays of* Query *(*Q*) and* Database* (*Db*) are transferred from CPU to GPU by using the following two libraries:

 
*cudaMemcpy(Db_d, Db, sizeof(struct Lingo)∗Db_Type, cudaMemcpyHostToDevice);*
 
*cudaMemcpy(Q_d, Q, sizeof(struct Lingo)∗Q_Type, cudaMemcpyHostToDevice).*

These two structure arrays are stored in the global memory. The time of transferring structure arrays from CPU to GPU is included in the time of* comparison phase*. A two-dimensional floating point array (result array) with a size of *k* × *r*, where *k* and *r* are the number of compounds in* Query* and* Database*, respectively, is created in the global memory. This array is used to store the computed Tanimoto coefficient for each comparison. For each comparison, the sorted LINGO scores of a compound in* Query* are used to compare with other sorted LINGO scores of a compound in* Database*. It is a simple job to find the same LINGO scores among these two compounds. By accumulating the LINGO numbers corresponding to the same LINGO scores, the Tanimoto coefficient of a comparison can be computed.

The pseudocode of computing Tanimoto coefficient is listed  in Pseudocode 2.

On multi-GPUs, the structure array of* Query* will be divided into several parts according to the computing capabilities of GPU cards. Then, these structure subarrays of* Query* are transferred from CPU to the global memory of the corresponding GPU, respectively. The complete structure array of* Database *also is transferred from CPU to the global memory of each GPU, respectively. For each GPU, a two-dimensional floating point array (partial result array) with a size of *m* × *r*, where *m* is the number of compounds in* Query* assigned to this GPU, is created in the global memory to store the computed Tanimoto coefficient for each comparison. The job of each comparison is similar to that on a single-GPU.

### 3.3. Output Phase

After the* comparison phase*, on single-GPU, the result array is transferred from GPU to CPU; on multi-GPUs, the partial result arrays are transferred from GPUs to CPU, respectively, and then these partial result arrays are merged into a complete result array. For each compound in* Query*, the compounds in* Database* with more than 0.85 Tanimoto coefficients are reported on CPU, respectively. The threshold 0.85 in CUDA-MCC is set up in order to report the compounds with the most (possible) similar structure. It can be adjusted to report the results according to the requirements, even for complete results.

The pseudocode of* output phase* is listed  in Pseudocode 3.

## 4. Experiment Results

In this work, CUDA-MCC was implemented by C+OpenMP+CUDA. In order to evaluate CUDA-MCC on single- and multi-GPUs, two machines are used in the experimental tests. The first machine has eight CPU cores; each core is Intel Xeon E5-2670 CPU of 2.6 GHz and single NVIDIA Tesla K20m GPU card with 2496 core of each 0.71 GHz. The second machine has eight CPU cores; each core is Intel Xeon E5-2650 CPU of 2.0 GHz and dual-NVIDIA Tesla K20m GPU card. There are three test sets used in the following tests: (s1) ten thousand compounds in* Query* and* Database*, respectively, (s2) thirty thousand compounds in* Query* and* Database*, respectively, and (s3) fifty thousand compounds in* Query* and* Database*, respectively. The test compounds are randomly selected from the ZINC database.

**Figure 3:**
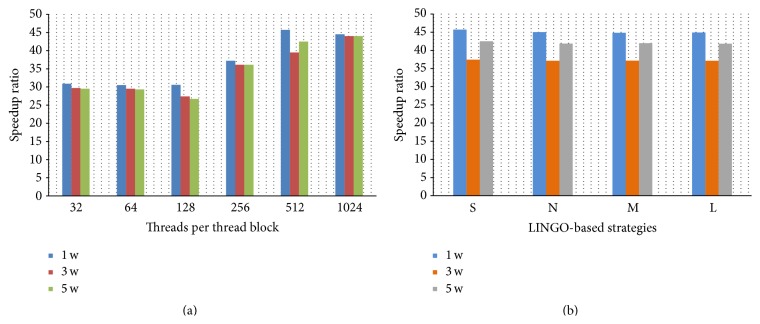
The speedup ratios for various numbers of threads in a thread block and four LINGO-based load-balancing strategies on three test sets and single NVIDIA Tesla K20m GPU card in machine 1.

The first test is to evaluate CUDA-MCC for these three test sets on single NVIDIA Tesla K20m GPU card in machines 1 and 2. Since the number of threads in a thread block will affect the performance by CUDA-MCC, various numbers of threads in a thread block are used in the tests. In addition, four LINGO-based load-balancing strategies are also applied into these three test sets by considering the LINGO score (denoted by S), LINGO number (denoted by N), LINGO length (denoted by L), and LINGO magnitude (denoted by M).  Figure 3(a) shows the speedup ratios of various numbers of threads in a thread block for these three test sets by CUDA-MCC on single NVIDIA Tesla K20m GPU card in machine 1. The speedup ratios by CUDA-MCC for four LINGO-based load-balancing strategies in these three test sets on single NVIDIA Tesla K20m GPU card in machine 1 are shown in Figure 3(b). In Figure 3(a), the speedup ratio increases when the number of threads in a thread block increases. Overall, 1024 threads in a thread block have the best speedup ratios among three test sets, and CUDA-MCC achieves 45 times faster than its CPU version on single NVIDIA Tesla K20m GPU card in machine 1. In Figure 3(b), CUDA-MCC achieves more than 35 times faster than its CPU version on single NVIDIA Tesla K20m GPU card in machine 1 for three test sets by each load-balancing strategy. The load-balancing strategies, S and M, outperform strategies L and N; however, the difference between them is very small. The performance and observations by CUDA-MCC on single NVIDIA Tesla K20m GPU card in machine 2 are similar to those in machine 1.

**Figure 4:**
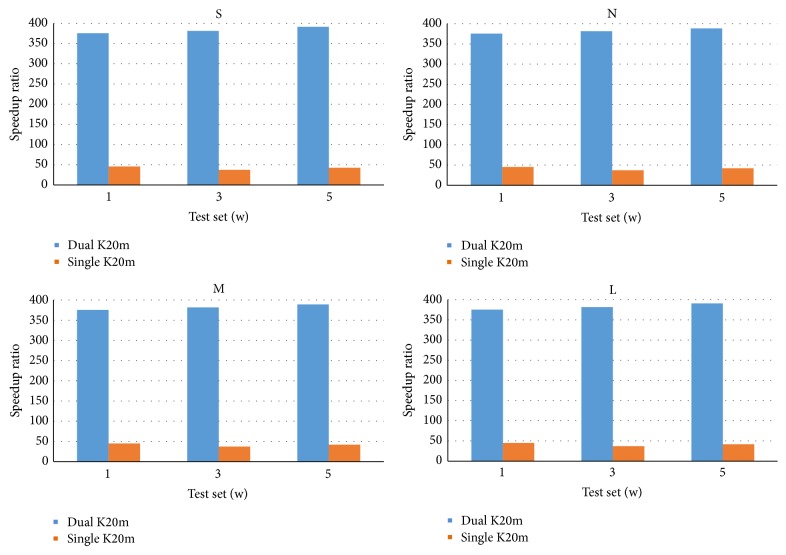
The comparisons of speedup ratios by CUDA-MCC on single NVIDIA Tesla K20m GPU card and dual-NVIDIA Tesla K20m GPU card in machine 2 for three test sets.

The second test is to evaluate CUDA-MCC for these three test sets on dual-NVIDIA Tesla K20m GPU card in machine 2. The goals of this test are to demonstrate that CUDA-MCC is useful for multi-GPUs, and the speedup ratios can be improved by using multi-GPU cards.  Figure 4 shows the comparisons of speedup ratios by CUDA-MCC on single NVIDIA Tesla K20m GPU card and dual-NVIDIA Tesla K20m GPU card in machine 2 for three test sets. From Figure 4, the speedup ratios by CUDA-MCC on dual-NVIDIA Tesla K20m GPU card in machine 2 are significantly larger than those by CUDA-MCC on single NVIDIA Tesla K20m GPU card in machine 2. CUDA-MCC achieves 391 times faster than its CPU version on dual-NVIDIA Tesla K20m GPU card in machine 2. The execution time of CUDA-MCC includes (*t*1) the time of transferring structure arrays (structure subarrays on multi-GPUs) from CPU to (corresponding) GPU in the* comparison phase*, (*t*2) the time of comparing two sets of compounds in the* comparison phase*, (*t*3) the time of transferring (partial result arrays on multi-GPUs) result array from (corresponding) GPU to CPU in the* output phase*, and (*t*4) the reporting (and the time to merge partial result arrays into a complete result array on multi-GPUs) time to report the compounds with similar structure. When running CUDA-MCC on multi-GPUs, both sizes of structure array in* Query* and result array, needed to be transferred between CPU and one GPU, reduce. Therefore, times *t*1 and *t*3 reduce greatly and the speedup ratio increases.

## 5. Conclusion

In this paper, a GPU-based algorithm, CUDA-MCC, was proposed and implemented to do the MCC problem on single- and multi-GPUs. Four LINGO-based load-balancing strategies were applied into CUDA-MCC, and then discuss the effects by considering the LINGO score, LINGO number, LINGO length, and LINGO magnitude, respectively. Two machines with single NVIDIA Tesla K20m GPU card and dual-NVIDIA Tesla K20m GPU card were used to evaluate CUDA-MCC, respectively. From experimental results, CUDA-MCC achieved 45 times and 391 times faster than its CPU version on single NVIDIA Tesla K20m GPU card and dual-NVIDIA Tesla K20m GPU card, respectively. Two observations were summarized in this work: (1) the speedup ratio increases when the number of threads in a thread block increases and (2) the LINGO score and LINGO magnitude strategies outperform other two strategies. However, the difference between them is very small. There are two possible reasons. First, the size of test sets is not enough to show the difference. Second, the benefits of estimating the computing workload by using these four LINGO types are the same. CUDA-MCC is useful for O2A and A2A comparisons on single- and multi-GPUs.

## Figures and Tables

**Pseudocode 1:**
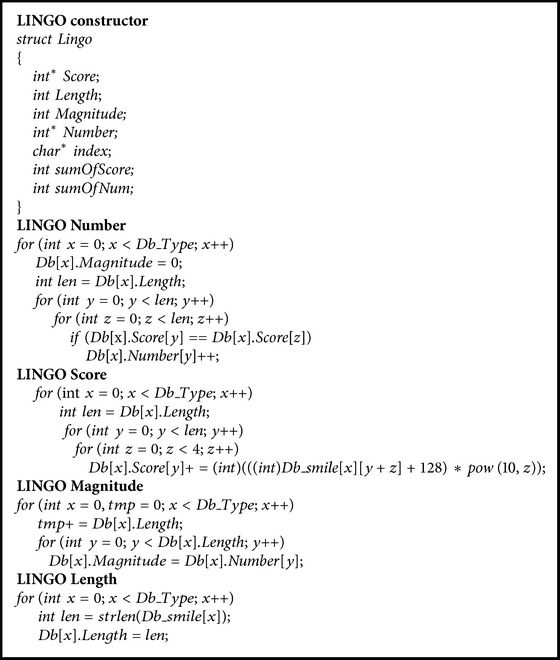
Pseudocodes of LINGO constructor, LINGO number, LINGO score, LINGO magnitude, and LINGO length.

**Pseudocode 2:**
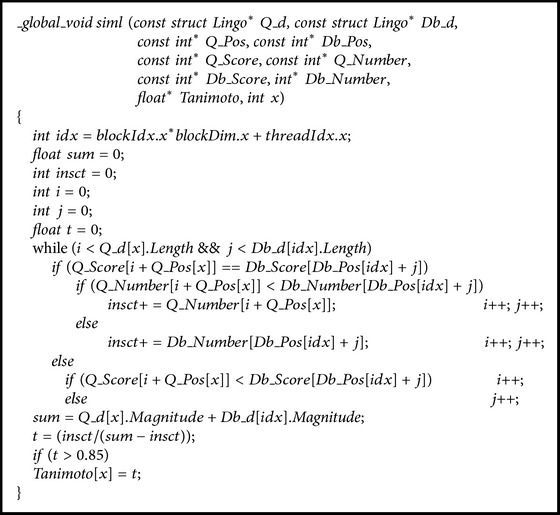
Pseudocode of computing Tanimoto coefficient.

**Pseudocode 3:**
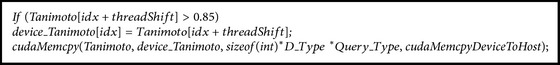
Pseudocode of output phase.
